# Hypotensive Anesthesia versus Normotensive Anesthesia during Major Maxillofacial Surgery: A Review of the Literature

**DOI:** 10.1155/2015/480728

**Published:** 2015-02-23

**Authors:** Michal Barak, Leiser Yoav, Imad Abu el-Naaj

**Affiliations:** ^1^The Department of Anesthesiology, Rambam Health Care Campus and the Bruce Rappaport Faculty of Medicine, Technion-Israel Institute of Technology, 31069 Haifa, Israel; ^2^The Department of Oral and Maxillofacial Surgery, Rambam Health Care Campus and the Bruce Rappaport Faculty of Medicine, Technion-Israel Institute of Technology, 31069 Haifa, Israel; ^3^The Department of Oral and Maxillofacial Surgery, Baruch Padeh Medical Center and the Faculty of Medicine, Bar-Ilan University, Poriya, 15208 Tiberias, Israel

## Abstract

Steady blood pressure within normal limits during surgery is one of the markers of the ideal and skillful anesthesia. Yet, reduced blood pressure is advantageous in some settings because it can contribute to a reduction in overall blood loss and improve the surgical field conditions. Controlled hypotension during anesthesia or hypotensive anesthesia is often used in major maxillofacial operations. Since hypotensive anesthesia carries the risk of hypoperfusion to important organs and tissues, mainly the brain, heart, and kidneys, it cannot be applied safely in all patients. In this paper we review the medical literature regarding hypotensive anesthesia during major maxillofacial surgery, the means to achieve it, and the risks and benefits of this technique, in comparison to normotensive anesthesia.

## 1. Introduction

Blood pressure is one of the essential vital signs that are monitored by health care professionals in modern medicine. In general, a normal blood pressure is an indicator of preserved cardiac output and good organ perfusion, and management of the patient often focuses on maintaining a normal blood pressure. Therefore, maintaining a patient's stable blood pressure within normal limits during surgery (normotensive anesthesia) is one of the indices of skillful anesthesia, and normotensive anesthesia is usually considered to be the gold standard for anesthesia.

The strategy of lowering the patient's blood pressure or controlled hypotension during anesthesia (hypotensive anesthesia) has been practiced for decades [1–5]. The physiological principle which underlies hypotensive anesthesia is a natural survival mechanism. When profuse bleeding occurs, the blood pressure drops. This drop leads to a reduction or cessation of the bleeding, blood pressure stabilization, and recovery. Accordingly, reducing the patient's blood pressure during surgery can potentially reduce overall bleeding. Since bleeding in the surgical field is also reduced, the surgical field operating conditions are improved.

The indications for hypotensive anesthesia are the surgical site, the course and extent of the surgery, and the patient's general condition. Hypotensive anesthesia is considered to be a suitable anesthetic technique for those patients who will be undergoing spinal surgery, hip or knee arthroplasty, craniosynostosis, hepatic resections, robotic surgery, and major maxillofacial operations [6–11]. However, the use of hypotensive anesthesia is associated with the risk of reduced perfusion to important organs and tissues, mainly the brain, heart, and kidneys [12, 13]. Thus, the hypotensive technique is potentially unsafe in some patients and is not suitable for all.

In this paper, we describe the means to achieve hypotensive anesthesia and compare the benefits and risks of hypotensive anesthesia and normotensive anesthesia during major maxillofacial operations.

## 2. Target Blood Pressure in Hypotensive Anesthesia

There are specific values that define normal blood pressure; nevertheless, “hypotension” is not an absolute term. Each individual has a range of blood pressures to which the individual's body is accustomed and within which it functions optimally. This range is considered “normal” for that individual. Thus, a patient whose blood pressure is usually at the lower range requires lower blood pressures than a hypertensive one. For normotensive anesthesia, the patient's blood pressure is maintained at levels that are within the range of blood pressure levels that were measured preoperatively. In hypotensive anesthesia, the patient's baseline mean arterial pressure (MAP) is reduced by 30% [14]. Consequently, the systolic blood pressure values are about 80–90 mm Hg and the MAP is reduced to 50–65 mm Hg [15].

## 3. Intraoperative Blood Loss in Normotensive and Hypotensive Anesthesia

Surgical procedures of the head and neck have a propensity to bleed profoundly because the region's blood supply is rich. These procedures are often extensive and prolonged thus inducing significant blood loss. The extent of intraoperative blood loss in normotensive and hypotensive anesthesia during maxillofacial operations has been compared in several clinical trials. Praveen et al. [16] conducted a prospective randomized clinical trial in which patients, who underwent orthognathic surgery, were randomly allocated to undergo these surgeries under normotensive or hypotensive anesthesia. They reported that the extent of intraoperative blood loss in those operations that were done under hypotensive anesthesia was substantially less than that in those operations that were done under normotensive anesthesia. These findings have been confirmed by others [10, 17–19]. Since intraoperative blood loss is reduced under hypotensive anesthesia, the need for allogeneic blood transfusion and its risks, namely, postoperative infection, acute lung injury, postoperative cardiac failure, tumor recurrence, perioperative myocardial infarction, and increased mortality [20], is also reduced.

## 4. Surgical Field Conditions in Normotensive and Hypotensive Anesthesia

Evaluation of the surgical field conditions is subjective and difficult to measure because the appraisal relies solely on the surgeon's assessment of the conditions. The duration of the surgery may be used as an objective indicator for surgical field conditions because the duration of an operation which is conducted under poor surgical field conditions may be longer than the one which is conducted under good surgical field conditions. The results of trials whose aim was to study the difference in surgical field conditions in major maxillofacial operations in hypotensive and normotensive anesthesia found that the surgical field conditions are better under hypotensive anesthesia than those under normotensive anesthesia despite the fact that there is no significant difference in the durations of the procedures [17, 21, 22].

## 5. Protocols for Hypotensive Anesthesia

Through the years, a multitude of drug combinations and protocols for hypotensive anesthesia have been suggested and compared. The two main strategies for achieving hypotensive anesthesia are (a) deep anesthesia and heavy analgesia and (b) standard anesthesia and administration of hypotensive drugs. By deepening the anesthetic plane and using high doses of analgesics, such as opioids, the recovery time may be prolonged. On the other hand, administering a hypotensive agent to a patient who is anesthetized using a standard anesthetic protocol may result in postoperative hypotension. In practice, the two strategies are used to achieve controlled hypotensive anesthesia. In the next section, we will discuss some of the anesthetic agents, analgesics, hypotensive drugs, and nonpharmacological methods that have been used for achieving hypotensive anesthesia.

### 5.1. Volatile Anesthetic Agents

Most anesthetic agents have a hypotensive effect: the blood pressure of a patient under general anesthesia is lower than that of same conscious patient. The volatile anesthetic agents, such as isoflurane, sevoflurane, and desflurane, have a potent vasodilator action, and this property can be exploited to reduce blood pressure by increasing the agent's concentration when administered to a patient. It has been reported that isoflurane, sevoflurane, and desflurane are each equal in their ability to reduce blood pressure [23, 24]. However, when volatile anesthetics are used alone, high concentrations are required to achieve a significant reduction in intraoperative bleeding, and these concentrations may lead to hepatic or renal injury. In addition, the volatile-mediated reduction in blood pressure is not meticulously controlled. The unwanted effects of these agents, such as nonthermoregulatory shivering and headaches, are to be expected during the postoperative period in patients recovering from isoflurane, sevoflurane, or desflurane anesthesia.

### 5.2. Propofol

Propofol, a widely used intravenous anesthetic agent, has a potent hypotensive capability. Accordingly, propofol has been used for achieving hypotensive anesthesia when administered as part of total intravenous anesthesia. Furthermore, normal blood pressure will be rapidly restored when the propofol infusion is discontinued. Although a short-term propofol infusion is safe, a long-term propofol infusion can cause propofol infusion syndrome in children [25, 26]. Ankichetty and colleagues compared using propofol to isoflurane for hypotensive anesthesia and found no significant difference in intraoperative blood loss and operative conditions [27]. Early postoperative complications following orthognathic operations that were conducted under hypotensive anesthesia were studied by Tabrizi and colleagues [28]. They found that total intravenous anesthesia using propofol offers no significant advantage over isoflurane-based anesthesia in terms of early postoperative complications, such as pain, nausea, vomiting, shivering, and agitation.

### 5.3. Alfentanil, Sufentanil, and Remifentanil

Alfentanil, sufentanil, and remifentanil are potent synthetic and short-acting opioid drugs of the anilidopiperidine family whose use has increased during the past three decades [29–32]. Alfentanil, a derivative of fentanyl, has a quicker onset and shorter duration of action than fentanyl and its vagomimetic properties are more intense than those of fentanyl and sufentanil. Sufentanil is a more potent analgesic than fentanyl and seems better than the other opioid analgesics, such as morphine or meperidine, in maintaining hemodynamic stability during surgery. Remifentanil is a potent mu-opioid receptor agonist that is rapidly metabolized by nonspecific blood and tissue esterases. According to its unique pharmacokinetic profile, remifentanil-based anesthesia combines high dose opioid intraoperative analgesia with a rapid and predictable postoperative awakening, which is independent of the duration of the infusion. When used for hypotensive anesthesia, each of these three drugs is equally effective in achieving hypotensive anesthesia for the required duration [33–35]. Since the recovery times from this type of anesthesia are also short, they are widely used for hypotensive anesthesia.

## 6. Hypotensive Drugs

Reducing blood pressure could be achieved in various ways that differ in the physiologic mechanism, duration, and side effects. The ideal hypotensive drug for inducing hypotensive anesthesia should be easy to administer, with a short onset time; its dose can be meticulously controlled; its effect disappears quickly when its administration is discontinued; it has a rapid elimination and causes no unwanted or adverse effects. In addition, it is important to match the drug with the patient's general condition, diseases, and daily medications.

Many hypotensive drugs with different mechanism and duration of action have been investigated for achieving hypotensive anesthesia [15]. These hypotensive drugs may be used alone or may be used in combination in order to limit the dose of each drug and minimize the occurrence of adverse effects of the other agents. The drugs that are used for hypotensive anesthesia include sodium nitroprusside (SNP), nitroglycerin (NTG), trimethaphan, calcium channel antagonists (e.g., nicardipine), *β*-adrenoceptor antagonists (e.g., propranolol and esmolol), angiotensin converting enzyme (ACE) inhibitors, and *α*_2_- adrenoceptor agonists (e.g., clonidine and dexmedetomidine). In addition to these agents, fenoldopam, adenosine, and alprostadil are new hypotensive drugs, which are currently being evaluated in settings that are not related to hypotensive anesthesia and are not yet in widespread clinical use. In the next section, we will discuss some of the hypotensive drugs that are commonly used in the protocols for hypotensive anesthesia.

### 6.1. Nitrates

SNP and NTG are two very potent hypotensive agents that are commonly used for inducing hypotensive anesthesia [15, 36]. The mechanism of their hypotensive action is rapid onset vigorous vasodilatation, which is mediated by nitric oxide. The main difference between SNP and NTG lies in their effect on the coronary blood flow. In addition, SNP is arterio- and venodilator, while NTG is mainly venodilator. Yoshikawa et al. [37] compared SNP and NTG administration for hypotensive anesthesia in patients who underwent mandibular osteotomy and reported that the extent of intraoperative blood loss is similar.

Both SNP and NTG can cause blood pressure to plunge following their intravenous administration due to a lowering of total peripheral resistance and/or venous return. The administration of SNP and NTG should be titrated carefully using a syringe pump because of the risk for accidental severe hypotension. The hypotensive action of nitrates can be quickly stopped by discontinuing its infusion. Reflex tachycardia is an unwanted effect which often occurs with nitrates administration and can be prevented by a small dose of a *β*-adrenoceptor antagonist, such as esmolol [38] or propranolol premedication [39].

### 6.2. *β*-Adrenoceptor Antagonists

The *β*-adrenoceptor antagonists have been effectively used for inducing hypotensive anesthesia for maxillofacial operations when administered either as a single hypotensive agent or in combination with SNP [37, 38, 40]. There are several *β*-antagonists in clinical use, and they differ in their duration of action and their selectivity for *β*-adrenoreceptors. The less selective *β*-antagonists, such as labetalol, may cause bronchoconstriction and are to be avoided in asthmatic patients [15]. The hypotensive action of *β*-adrenoceptor antagonists is achieved by reducing cardiac output. Accordingly, these drugs are not suitable for patient with underlying heart failure. When administration of the drug is stopped, reflex tachycardia can occur.

### 6.3. Calcium Channel Antagonists

Calcium channel antagonists, such as nifedipine or nicardipine, are commonly used hypotensive drugs. Kim and others [41] tested the hypothesis that the adverse effects of hypotensive anesthesia on renal function can be prevented by a continuous nicardipine infusion. In order to test this hypothesis, they measured the blood levels of biomarkers for subclinical and reversible renal dysfunction that appear during hypotensive anesthesia for orthognathic surgery that was induced by either a continuous nicardipine infusion or a combination of desflurane-induced anesthesia and remifentanil. They found that both anesthetic protocols increased the blood levels of the biomarkers, though the increase was less in those patients to whom nicardipine was administered [41].

## 7. Nonpharmacological Means for Achieving Hypotension

### 7.1. The Anti-Trendelenburg Position

Hypotension in the anesthetized patient can be easily achieved by placing the patient in a head-up or anti-Trendelenburg position because of orthostatic or postural hypotension results. This positioning is frequently used for hypotensive anesthesia and normal blood pressure can be quickly restored by repositioning the patient [42]. When using this method, one should remember that the patient's response to the head-up tilt is inconsistent and depends on the patient's cardiac output [43].

### 7.2. Acute Normovolemic Hemodilution (ANH)

ANH is accomplished by drawing a unit or two of the patient's blood either immediately before or shortly after the induction of anesthesia and simultaneously replacing it with a cell-free fluid, preferably a synthetic colloid solution [44, 45]. In this setting, the patient bleeds “diluted” blood (the number of red blood cells in the blood is reduced), and upon completion of the surgery, the autologous blood is retransfused back to the patient. Consequently, when the patient bleeds during the operation, the volume of red blood cell loss is decreased. ANH is to be considered in patients who (a) are undergoing major elective surgery, (b) presenting with an initial hemoglobin concentration which is greater or equal to 12 g/dL, and (c) will have an anticipated blood loss of more than or equal to 1500 mL [43]. ANH has been used for decades in major operations [46–48] and is considered to be a part of a blood conservation strategy which reduces or limits the need for allogeneic blood transfusion during surgery [49, 50]. Adverse effects which can occur during ANH are hemodynamic changes, such as a decreased cardiac output [51, 52].

Although ANH can be a part of normotensive or hypotensive anesthesia, the procedure often results in a reduced blood pressure. Ervens et al. compared three anesthetic protocols, namely, normotensive anesthesia, hypotensive anesthesia, and hypotensive anesthesia combined with ANH, in a surgeon-blinded trial of 60 patients who required either a Le Fort I osteotomy or a bimaxillary surgery [18]. They reported that the extent of intraoperative blood loss and the requirements for an allogeneic blood transfusion were substantially reduced in those patients who underwent hypotensive anesthesia. They also reported that hypotensive anesthesia combined with ANH had no additional blood-sparing effects or surgical field quality improvement in orthognathic surgery.

## 8. Patient Selection for Hypotensive Anesthesia

Hypotensive anesthesia is not suitable for all patients because reducing the blood pressure is potentially unsafe in some patients. Patients with disseminated vascular disease, in whom atheromatous vessels provide perfusion to the organs, may suffer from hypoperfusion during hypotension, with the brain and heart at the highest danger to be injured. Patients with ischemic heart disease [53, 54] or carotid artery stenosis [55, 56] are at highest risk for clinically significant hypotension-induced injury following hypotensive anesthesia. These patients are candidates for normotensive anesthesia or, in selective cases, minor reduction in blood pressure, the so-called “modified hypotensive anesthesia”, adjusted to their condition.

Another group of patients whose risks of hypotensive anesthesia are high are hypertensive patients, because the functions of their vital organs are adjusted to their usual high blood pressure. These patients are sensitive to reduced pressure and are at high risk for the occurrence of perioperative complications. In addition, hypertensive patients often have contracted blood volume and are extremely susceptible to vasodilatation [57]. If hypotensive anesthesia is to be used in a hypertensive patient, the patient should be closely monitored and managed with great caution because of the risk of a profound and rapid decrease in blood pressure due to the action of the hypotensive drugs. In addition, the patient should be given appropriate volume replacement prior to commencing hypotensive anesthesia.

Reduced blood pressure during surgery may result in transient tubular dysfunction in patients with normal preoperative renal function [35], and the use of hypotensive anesthesia may exacerbate renal function in patients with a known preoperative kidney disease. It is important to remember that the renal functions are affected mainly by the cardiac output and the blood flow in the splanchnic circulation and not by systemic arterial pressure. Thus, blood pressure measurements may not accurately indicate the actual renal perfusion pressure. In the study that we previously cited [41] the authors found that the use of nicardipine, a calcium channel antagonist, had a subtle protective effect on the kidneys during hypotensive anesthesia for maxillofacial surgery.

In the future, we anticipate studying new drugs which will induce hypotension without impairing the perfusion and function of vital organs.

## 9. Type of Maxillofacial Operation

Operations in the maxillofacial region require precise, accurate, and delicate surgery of the hard and soft tissues. The head and neck region is abundantly vascularized; this is a great advantage regarding healing and regeneration; yet it can be a major cause of severe life threatening bleeding during surgery. Several operations are at increased risk for extensive bleeding, including Le Fort osteotomies [58–60], maxillectomy for tumor resection, tumor resection from the tongue and floor of the mouth, and neck dissections [4, 5]. In all these procedures, hypotensive anesthesia is needed in order to reduce intraoperative bleeding in the surgical field, maintain the surgical plane, avoid unnecessary damage to the vital structures and tissues, and execute the required surgical procedure. It is important to identify procedures in which high blood loss is expected and restrict hypotensive anesthesia technique usage to these procedures and for the patients who are most likely to benefit by that.

### 9.1. Hypotensive Anesthesia and Maxillofacial Trauma

The use of hypotensive anesthesia in trauma patients is relatively new and controversial [61, 62]. In urgent or emergent operations of trauma patients, where there is major trauma to the face and neck, severe uncontrolled bleeding is possible, mostly in surgeries that involve more than two thirds of the face, panfacial trauma. Naturally, in a longer and more extensive procedure, the bleeding is more pronounced and controlling it is more difficult. In such cases the application of hypotensive anesthesia may be useful. Nonetheless, trauma patients with facial injury often have other injuries, such as head trauma. A hypotensive approach may limit further bleeding but could aggravate any existing brain injury. The management of these patients, including their target blood pressure, should take into account all involved injuries. The ideal approach to these complex patients requires further investigation.

## 10. Conclusions

Patients who undergo major maxillofacial surgery are at risk of considerable intraoperative bleeding, and the outcome of the surgical procedure depends on the quality of the surgical field conditions. Since hypotensive anesthesia can reduce the extent of intraoperative bleeding and can potentially improve the quality of the surgical field conditions, hypotensive anesthesia is considered to be beneficial during these procedures. However, hypotension carries the risk of hypoperfusion in vital organs and is unsafe in certain patients. Thus, the magnitude of the blood pressure reduction should be adjusted to the patient's general condition, age, and existing diseases. Normotensive or modified hypotensive anesthesia should be used for patients with ischemic heart disease, carotid artery stenosis, disseminated vascular disease, kidney dysfunction, or severe hypertension who are scheduled to undergo a major maxillofacial operation.

Appropriate patient selection, careful monitoring, and adequate intraoperative volume replacement are mandatory in hypotensive anesthesia for its safe implementation in patients who are scheduled to undergo a major maxillofacial operation.

## References

[1] Bentel H. (1968). Hypotensive anaesthesia in head and neck surgery. *Diastema*.

[2] Warner W. A., Shumrick D. A., Caffrey J. A. (1970). Clinical investigation of prolonged induced hypotension in head and neck surgery. *British Journal of Anaesthesia*.

[3] Mostert J. W. (1973). Safe hypotensive anesthesia. *The Journal of the American Medical Association*.

[4] Sataloff R. T., Brown A. C. D., Sheets E. E., Rubinstein M. I. (1987). A controlled study of hypotensive anesthesia in head and neck surgery. *Ear, Nose and Throat Journal*.

[5] Ward C. F., Alfery D. D., Saidman L. J., Waldman J. (1980). Deliberate hypotension in head and neck surgery. *Head and Neck Surgery*.

[6] Hassan N., Halanski M., Wincek J. (2011). Blood management in pediatric spinal deformity surgery: review of a 2-year experience. *Transfusion*.

[7] Banerjee S., Issa K., Kapadia B. H. (2013). Intraoperative nonpharmacotherapeutic blood management strategies in total knee arthroplasty. *Journal of Knee Surgery*.

[8] Fearon J. A., Cook T. K., Herbert M. (2014). Effects of hypotensive anesthesia on blood transfusion rates in craniosynostosis corrections. *Plastic and Reconstructive Surgery*.

[9] Papalia R., Simone G., Ferriero M. (2012). Laparoscopic and robotic partial nephrectomy with controlled hypotensive anesthesia to avoid hilar clamping: feasibility, safety and perioperative functional outcomes. *Journal of Urology*.

[10] Piñeiro-Aguilar A., Somoza-Martin M., Gandara-Rey J. M., García-García A. (2011). Blood loss in orthognathic surgery: a systematic review. *Journal of Oral and Maxillofacial Surgery*.

[11] Chen C. M., Lai S. S., Hsu K. J., Lee H. E., Huang H. L. (2011). Assessment of the related factors of blood loss and blood ingredients among patients under hypotensive anesthesia in orthognathic surgery. *Journal of Craniofacial Surgery*.

[12] Strunin L. (1975). Organ perfusion during controlled hypotension. *British Journal of Anaesthesia*.

[13] Lindop M. J. (1975). Complications and morbidity of controlled hypotension. *The British Journal of Anaesthesia*.

[14] Rodrigo C. (1995). Induced hypotension during anesthesia, with special reference to orthognathic surgery. *Anesthesia Progress*.

[15] Degoute C.-S. (2007). Controlled hypotension: a guide to drug choice. *Drugs*.

[16] Praveen K., Narayanan V., Muthusekhar M. R., Baig M. F. (2001). Hypotensive anaesthesia and blood loss in orthognathic surgery: a clinical study. *British Journal of Oral and Maxillofacial Surgery*.

[17] Dolman R. M., Bentley K. C., Head T. W., English M. (2000). The effect of hypotensive anesthesia on blood loss and operative time during Le Fort I osteotomies. *Journal of Oral and Maxillofacial Surgery*.

[18] Ervens J., Marks C., Hechler M., Plath T., Hansen D., Hoffmeister B. (2010). Effect of induced hypotensive anaesthesia vs isovolaemic haemodilution on blood loss and transfusion requirements in orthognathic surgery: a prospective, single-blinded, randomized, controlled clinical study. *International Journal of Oral and Maxillofacial Surgery*.

[19] Varol A., Basa S., Ozturk S. (2010). The role of controlled hypotension upon transfusion requirement during maxillary downfracture in double-jaw surgery. *Journal of Cranio-Maxillofacial Surgery*.

[20] Ashworth A., Klein A. A. (2010). Cell salvage as part of a blood conservation strategy in anaesthesia. *The British Journal of Anaesthesia*.

[21] Precious D. S., Splinter W., Bosco D. (1996). Induced hypotensive anesthesia for adolescent orthognathic surgery patients. *Journal of Oral and Maxillofacial Surgery*.

[22] Carlos E., Monnazzi M. S., Castiglia Y. M., Gabrielli M. F., Passeri L. A., Guimarães N. C. (2014). Orthognathic surgery with or without induced hypotension. *International Journal of Oral and Maxillofacial Surgery*.

[23] Dal D., Çeliker V., Özer E., Başgül E., Salman M. A., Aypar Ü. (2004). Induced hypotension for tympanoplasty: a comparison of desflurane, isoflurane and sevoflurane. *European Journal of Anaesthesiology*.

[24] Rossi A., Falzetti G., Donati A., Orsetti G., Pelaia P. (2010). Desflurane versus sevoflurane to reduce blood loss in maxillofacial surgery. *Journal of Oral and Maxillofacial Surgery*.

[25] Hanna J. P., Ramundo M. L. (1998). Rhabdomyolysis and hypoxia associated with prolonged propofol infusion in children. *Neurology*.

[26] Patermann B., Buzello S., Dück M., Paul M., Kampe S. (2004). Accidental tenfold overdose of propofol in a 6-month old infant undergoing elective craniosynostosis repair. *Anaesthesia*.

[27] Ankichetty S. P., Ponniah M., Cherian V. T. (2011). Comparison of total intravenous anesthesia using propofol and inhalational anesthesia using isoflurane for controlled hypotension in functional endoscopic sinus surgery. *Journal of Anaesthesiology Clinical Pharmacology*.

[28] Tabrizi R., Eftekharian H. R., Langner N. J., Ozkan B. T. (2012). Comparison of the effect of 2 hypotensive anesthetic techniques on early recovery complications after orthognathic surgery. *Journal of Craniofacial Surgery*.

[29] Reitz J. A. (1986). Alfentanil in anesthesia and analgesia. *Drug Intelligence & Clinical Pharmacy*.

[30] Monk J. P., Beresford R., Ward A. (1988). Sufentanil: a review of its pharmacological properties and therapeutic use. *Drugs*.

[31] Beers R., Camporesi E. (2004). Remifentanil update: clinical science and utility. *CNS Drugs*.

[32] Servin F. S., Billard V. (2008). Remifentanil and other opioids.. *Handbook of Experimental Pharmacology*.

[33] Farah G. J., de Moraes M., Filho L. I. (2008). Induced hypotension in orthognathic surgery: a comparative study of 2 pharmacological protocols. *Journal of Oral and Maxillofacial Surgery*.

[34] Uzun S., Yuce Y., Erden A., Aypar U. (2014). Impact of perioperative lidocaine infusion and bis monitorization on remifentanil dosage in hypotensive anesthesia. *European Review for Medical and Pharmacological Sciences*.

[35] Lubrano R., Marandola M., Antonucci A. (2008). Hypotensive anesthesia with propofol and remifentanil: protective effect of alpha-tocopherol on renal function. *Journal of Clinical Anesthesia*.

[36] Choi S. H., Lee S. J., Jung Y. S. (2008). Nitroglycerin- and nicardipine -induced hypotension does not affect cerebral oxygen saturation and postoperative cognitive function in patients undergoing orthognathic surgery. *Journal of Oral and Maxillofacial Surgery*.

[37] Yoshikawa F., Kohase H., Umino M., Fukayama H. (2009). Blood loss and endocrine responses in hypotensive anaesthesia with sodium nitroprusside and nitroglycerin for mandibular osteotomy. *International Journal of Oral and Maxillofacial Surgery*.

[38] Hanamoto H., Sugimura M., Morimoto Y., Kudo C., Boku A., Niwa H. (2012). Small bolus of esmolol effectively prevents sodium nitroprusside-induced reflex tachycardia without adversely affecting blood pressure. *Journal of Oral and Maxillofacial Surgery*.

[39] Apipan B., Rummasak D. (2010). Efficacy and safety of oral propranolol premedication to reduce reflex tachycardia during hypotensive anesthesia with sodium nitroprusside in orthognathic surgery: a double-blind randomized clinical trial. *Journal of Oral and Maxillofacial Surgery*.

[40] McNulty S., Sharifi-Azad S., Farole A. (1987). Induced hypotension with labetalol for orthognathic surgery. *Journal of Oral and Maxillofacial Surgery*.

[41] Kim J. E., Lee J. S., Kim M. K., Kim S. H., Kim J. Y. (2014). Nicardipine infusion for hypotensive anesthesia during orthognathic surgery has protective effect on renal function. *Journal of Oral and Maxillofacial Surgery*.

[42] Rodrigo C. (2000). Anesthetic considerations for orthognathic surgery with evaluation of difficult intubation and technique for hypotensive anesthesia. *Anesthesia Progress*.

[43] Stefadouros M. A., El Shahawy M., Stefadouros F., Witham A. C. (1975). The effect of upright tilt on the volume of the failing human left ventricle. *The American Heart Journal*.

[44] Kreimeier U., Messmer K. (2002). Perioperative hemodilution. *Transfusion and Apheresis Science*.

[45] Monk T. G. (2005). Acute normovolemic hemodilution. *Anesthesiology Clinics of North America*.

[46] Casati V., Speziali G., D'Alessandro C. (2002). Intraoperative low-volume acute normovolemic hemodilution in adult open-heart surgery. *Anesthesiology*.

[47] Oppitz P. P., Stefani M. A. (2013). Acute normovolemic hemodilution is safe in neurosurgery. *World Neurosurgery*.

[48] Jarnagin W. R., Gonen M., Maithel S. K. (2008). A prospective randomized trial of acute normovolemic hemodilution compared to standard intraoperative management in patients undergoing major hepatic resection. *Annals of Surgery*.

[49] Bennett J., Haynes S., Torella F., Grainger H., McCollum C. (2006). Acute normovolemic hemodilution in moderate blood loss surgery: a randomized controlled trial. *Transfusion*.

[50] Shander A., Rijhwani T. S. (2004). Acute normovolemic hemodilution. *Transfusion*.

[51] Kungys G., Rose D. D., Fleming N. W. (2009). Stroke volume variation during acute normovolemic hemodilution. *Anesthesia and Analgesia*.

[52] Licker M., Ellenberger C., Sierra J., Christenson J., Diaper J., Morel D. (2005). Cardiovascular response to acute normovolemic hemodilution in patients with coronary artery diseases: assessment with transesophageal echocardiography. *Critical Care Medicine*.

[53] Choi W. S., Samman N. (2008). Risks and benefits of deliberate hypotension in anaesthesia: a systematic review. *International Journal of Oral and Maxillofacial Surgery*.

[54] Singh A., Antognini J. (2011). Perioperative hypotension and myocardial ischemia: diagnostic and therapeutic approaches. *Annals of Cardiac Anaesthesia*.

[55] Ruff R. L., Talman W. T., Petito F. (1981). Transient ischemic attacks associated with hypotension in hypertensive patients with carotid artery stenosis. *Stroke*.

[56] Wong D. H. W. (1991). Perioperative stroke. Part I: general surgery, carotid artery disease, and carotid endarterectomy. *Canadian Journal of Anaesthesia*.

[57] Seo H. S., Kim E. J., Kim S. W. (2013). Extracellular fluid adjusted for body size is contracted in hypertension. *Hypertension Research*.

[58] Dhariwal D. K., Gibbons A. J., Kittur M. A., Sugar A. W. (2004). Blood transfusion requirements in bimaxillary osteotomies. *British Journal of Oral and Maxillofacial Surgery*.

[59] Kretschmer W. B., Baciut G., Bacuit M., Zoder W., Wangerin K. (2010). Intraoperative blood loss in bimaxillary orthognathic surgery with multisegmental Le Fort I osteotomies and additional procedures. *British Journal of Oral and Maxillofacial Surgery*.

[60] Rummasak D., Apipan B., Kaewpradup P. (2011). Factors that determine intraoperative blood loss in bimaxillary osteotomies and the need for preoperative blood preparation. *Journal of Oral and Maxillofacial Surgery*.

[61] Tobin J. M., Dutton R. P., Pittet J. F., Sharma D. (2014). Hypotensive resuscitation in a head-injured multi-trauma patient. *Journal of Critical Care*.

[62] Harris T., Davenport R., Hurst T., Jones J. (2012). Improving outcome in severe trauma: trauma systems and initial management-intubation, ventilation and resuscitation. *Postgraduate Medical Journal*.

